# Distracting Through Procedural Pain and Distress Using Virtual Reality and Guided Imagery in Pediatric, Adolescent, and Young Adult Patients: Randomized Controlled Trial

**DOI:** 10.2196/30260

**Published:** 2022-04-18

**Authors:** Jennifer A Hoag, Jeffrey Karst, Kristin Bingen, Akasha Palou-Torres, Ke Yan

**Affiliations:** 1 Division of Pediatric Psychology and Developmental Medicine Department of Pediatrics Medical College of Wisconsin Milwaukee, WI United States

**Keywords:** virtual reality, procedural, pain, anxiety, pediatric, guided imagery

## Abstract

**Background:**

Children with acute and chronic illness undergo frequent, painful, and distressing procedures.

**Objective:**

This randomized controlled trial was used to evaluate the effectiveness of guided imagery (GI) versus virtual reality (VR) on the procedural pain and state anxiety of children and young adults undergoing unsedated procedures. We explored the role of trait anxiety and pain catastrophizing in intervention response.

**Methods:**

Children and young adults were recruited from the hematology, oncology, and blood and marrow transplant clinics at a children’s hospital. Each study participant completed the GI and VR intervention during separate but consecutive unsedated procedures. Self-report measures of pain and anxiety were completed before and after the procedures.

**Results:**

A total of 50 participants (median age 13 years) completed both interventions. GI and VR performed similarly in the management of procedural pain. Those with high pain catastrophizing reported experiencing less nervousness about pain during procedures that used VR than those using GI. State anxiety declined pre- to postprocedure in both interventions; however, the decrease reached the level of significance during the VR intervention only. Those with high trait anxiety had less pain during GI.

**Conclusions:**

In our sample, VR worked as well as GI to manage the pain and distress associated with common procedures experienced by children with acute or chronic illnesses. Children who are primed for pain based on beliefs about pain or because of their history of chronic pain had a better response to VR. GI was a better intervention for those with high trait anxiety.

**Trial Registration:**

ClinicalTrials.gov NCT04892160; https://clinicaltrials.gov/ct2/show/NCT04892160

## Introduction

### Pain

According to the International Association for the Study of Pain, “pain is always a personal experience that is influenced to varying degrees by biological, psychological, and social factors” [1]. Studies estimate that as many as half of children with acute or chronic illnesses experience procedure-related pain and distress [2]. In the short term, pain can manifest as withdrawal, clinginess, moodiness, or anger. There is substantial evidence, however, that inadequately addressed pain in childhood is associated with neurological and behavioral outcomes, including increased pain sensitivity, over the life course [3]. For example, children with sickle cell disease with a higher frequency of painful vaso-occlusive episodes are more likely to report heightened pain responses during venipuncture [4].

Children with cancer, sickle cell disease, and other blood disorders undergo routine procedures over many months or years. Unsurprisingly, pain from diagnostic procedures and treatment is one of the most frequently cited physical problems in children undergoing cancer treatment [5]. Over the past 40 years, there has been a trend toward increased pain control through the use of sedation and analgesia; however, there are risks to sedation, including hypoxia, that outweigh the benefits in recurrent and routine procedures. The identification and use of nonpharmacological interventions to manage pain could mitigate the risk of neurological and behavioral changes that result from poorly managed pain without the risks of sedation.

### Guided Imagery

Distraction is an effective and readily available nonpharmacologic tool for pain management [6,7]. It suppresses the highly salient sensations of pain and anxiety by consciously shifting attention to a more pleasant activity or thought. Guided imagery (GI) is a powerful nonimmersive distraction that involves describing in detail a situation incompatible with the experience of pain and is meant to evoke feelings of calm. GI scripts often begin with brief relaxation exercises, such as diaphragmatic breathing, followed by a vivid description of a relaxing activity, such as walking along a beach, flying among the clouds, or participating in a campfire. It is widely regarded as useful in decreasing pain and anxiety during procedures that do not warrant pharmacologic intervention [8-11].

### Virtual Reality

Virtual reality (VR) is an immersive, 3D, interactive technology that engages multiple senses and creates an artificial environment that the user can inhabit. VR has been used to assist with pediatric procedural distress in several contexts over the past 20 years such as burn care [12,13], dental procedures [14-16], intravenous needle sticks [17-25], and port access [26-28]. Reviews of VR use have been positive, with most suggesting that VR is a feasible and efficacious method of distraction that can reduce patient-reported pain and distress [29].

While there have been numerous studies comparing VR to no intervention [20,28] and VR to standard of care (primarily access to television or tablets [17,21,22]), there have been no studies directly comparing the widely accepted nonimmersive distraction of GI to the promising immersive distraction of VR. Since procedure-related pain cannot be avoided, it is important to investigate which intervention provides the most relief and whether there are subcategories of children who respond better to one intervention over another. For example, research has demonstrated that individuals who are primed for pain and hold catastrophic beliefs about pain have more difficulty being distracted during painful experiences [30-32]. Similarly, state anxiety, a fluid variable that describes one’s current level of anxiety, is predictive of pain tolerance and pain-related anxiety [33]. In their experiment using noxious electrical stimuli, Tang and Gibson [34] found that even when state anxiety was lower, individuals with high trait anxiety (ie, a stable variable that indicates greater disposition to experience anxiety) still reported higher subjective pain intensity ratings than those with low trait anxiety. Johnson [35] posited that the more distracting the stimuli, the greater the reduction in an individual’s capacity to process pain and feel distressed. There is also evidence that active distraction techniques are more beneficial for pain management than passive approaches [36].

In this study, we directly compare the effects of VR and GI during an unsedated procedure on subjective and objective measures of pain and anxiety. We hypothesize that the VR intervention will be associated with decreased experiences of procedural pain and distress as compared to the nonimmersive GI intervention. We further hypothesize that the impact of VR on reducing procedural pain and anxiety will be greater in pediatric patients who have higher levels of pain catastrophizing and greater state and trait anxiety.

## Methods

### Study Design

This was a single-site, crossover, randomized controlled trial (RCT) used to evaluate the effectiveness of GI versus VR on the procedural pain and distress of children and young adults undergoing unsedated procedures. A convenience sample of participants was recruited from the hematology, oncology, and blood and marrow transplant services at a large tertiary children’s hospital in Wisconsin. Data were collected between February 2018 and April 2019, at which point the threshold of enrolled patients had been reached. The interventions included (1) nonimmersive distraction via a 15-minute audio recording of a guided imagery script and (2) immersive distraction using KindVR Aqua (KindVR LLC), a virtual reality game that runs over 15 minutes. Conditions were counterbalanced so that there were 2 possible condition orders (VR/GI and GI/VR).

### Participants

**Figure 1:**
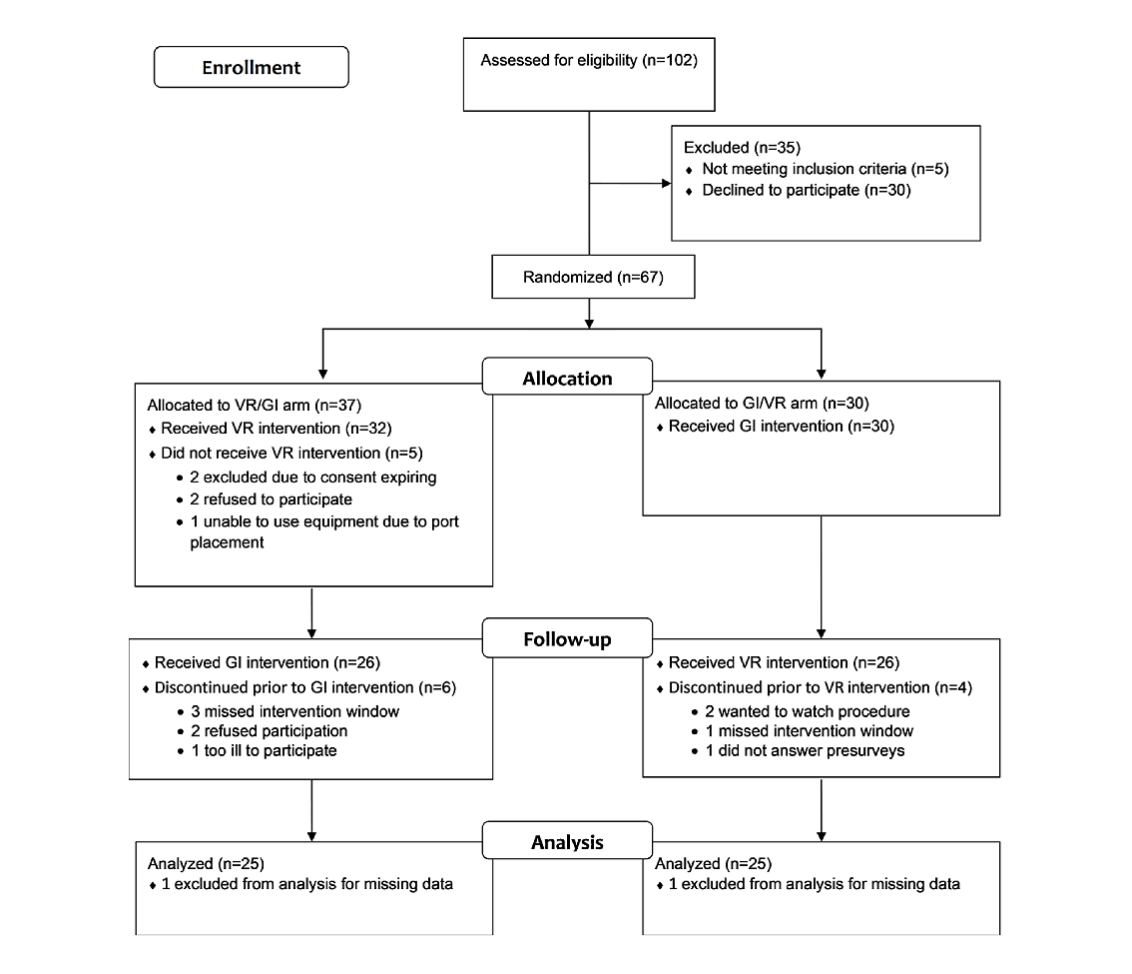
Consolidated Standards on Reporting Trials flow diagram. GI: guided imagery; VR: virtual reality.

Children and young adults aged 8 to 25 years seen by the hematology, oncology, or blood and marrow transplant services were eligible if they were at least 1-month postdiagnosis and undergoing one of the following unsedated procedures: venipuncture, port access, or peripherally inserted central-line catheter or central venous line dressing change. Patients were excluded if they were not able to read or speak English proficiently, had identified physical impairments (eg, blindness, active infection of the skin, history of seizure disorder) that would have prevented them from using VR equipment, or had significant developmental delays that would have prevented them from completing required study questionnaires. There were 102 children and young adults screened for eligibility. Of these, 34.3% (35/102) were excluded for the reasons indicated in the CONSORT (Consolidated Standards on Reporting Trials) flow diagram (Figure 1). Of the remaining 67 participants, 37 were randomized into the VR/GI arm and 30 were randomized into the GI/VR arm. A total of 52 participants completed both interventions, and 2 were excluded from the final analysis due to missing data, resulting in a final sample of 50 participants.

Study personnel identified eligible patients via the clinic schedule and inpatient census. After enrollment, study participants were randomly assigned to one of 2 possible condition orders using an online random number generator [37]. All attempts were made to ensure the study conditions (VR, GI) took place over the course of 2 consecutive procedures, excluding unplanned or emergent procedures. Conditions were separated by a minimum of 5 days and a maximum of 40 days to minimize the threat of treatment artifacts and extraneous events. The minimum time limit ensured that patients would not participate in 2 conditions within the same calendar week, while the maximum time limit allowed participation of patients who receive treatment on approximately a monthly basis. Both time points for an individual participant involved the same procedure type (ie, venipuncture, port access, or dressing change). Participants completed preprocedure questionnaires and, 3 to 5 minutes before the nurse entered the room for the procedure, the intervention was started. Each intervention lasted approximately 15 minutes. At the end of the procedure, participants completed their postprocedure questionnaires. Study personnel remained in the room to provide technical assistance and complete an observational measure of distress (ie, Children’s Emotional Manifestation Scale [CEMS]).

### Interventions

#### VR Condition

The VR intervention consisted of an interactive audio and visual underwater experience. The VR software used was KindVR Aqua, a research-based game focused on reducing pain and distress during medical procedures. Aqua offered both passive and active gameplay. In the passive experience, the software moved participants through an ocean filled with sea creatures and allowed them to observe the underwater scene. In the active experience, participants launched balls at the sea creatures. When hit, the creatures turned a variety of bright colors and points were earned. Participants were encouraged but not required to actively participate to increase the level of distraction. Study personnel recorded whether the participant participated in the active portion of the VR program. We used an off-the-shelf consumer headset (Gear VR, Samsung) powered by a Samsung smartphone with over-ear, noise-cancelling headphones. A wireless controller was used to interact with the underwater environment; the controller could be used with one hand if the procedure necessitated. The equipment did not require internet capability.

#### GI Condition

The GI script used in this study described an underwater scene that closely mimicked the VR condition. Similar to other GI scripts, ours began with instructions to take a few deep breaths to aid in relaxation. We then offered vivid descriptions of swimming underwater, which were similar to those seen in the VR intervention (eg, “You hear off in the distance the faint, yet beautiful songs of friendly whales talking to one another while making their way through the clear blue water. A sea turtle then glides past you. His face and flippers are patterned with spots of deep tan and brown, reflecting the rays of sunlight streaming through the water”). The script was audiorecorded on a tablet. Participants listened to the recording using over-ear, noise-cancelling headphones to approximate the headphones used during the VR condition.

### Measures

Patient perceptions of pain were assessed prior to the first procedure using either the child (for participants aged 8-16 years) or adult (for participants aged 17 years and older) version of the Pain Catastrophizing Scale (PCS) [38]. The PCS is a 13-question survey that assesses thoughts and feelings related to pain, specifically catastrophic thinking about pain, on a 5-point scale ranging from 1 (not at all) to 5 (extremely). The measure includes a total score and rumination, magnification, and helplessness subscales. Scores range from 0 to 52 with scores >30 considered to indicate an elevated level of catastrophic thinking.

A visual analog scale (VAS) was used to assess pain and distress after each procedure. This measure asked about 4 domains of pain, including worst pain, average amount of pain, nervousness about pain, and time spent thinking about pain [39]. Scores range from 0 to 100 with higher scores indicating worsening symptoms.

The CEMS [40] was completed by study personnel during each of the study visits. This observational measure offers an objective way to measure distress during difficult medical experiences. It includes the following domains: anxiety score, facial expression, vocalization, activity, interaction, and level of cooperation. Each domain is scored using a 5-point scale. Total scores range from 1 to 25 with higher scores indicating more distress.

Trait (underlying or baseline) anxiety was assessed prior to the first procedure using the trait portion of either the child (for participants aged 8-16 years) or adult (for participants aged 17 years and older) version of the State-Trait Anxiety Inventory (STAI), while state (in the moment) anxiety was assessed prior to and following each procedure using the state portion of the STAI [41]. The STAI includes 40 items in 2 subscales (state and trait anxiety). Subscale scores for children range from 20 to 60, and scores for adults range from 20 to 80. Higher scores indicate greater anxiety. Scores were transformed to z-scores for the purposes of comparison.

A demographic survey was completed by the patient or patient’s caregiver prior to study completion. Relevant treatment history was collected from the patient’s medical record. This information included date of birth, diagnosis date, dates of procedures, treatment type, and relapse status. All dates were removed once calculations were made (eg, time between visits, age at diagnosis).

### Ethical Approval

The institutional review board at Children’s Wisconsin reviewed and approved all study documents and protocols (1110230-13), and the study was registered at ClinicalTrials.gov [NCT04892160]. All study participants and their caregivers were informed about the study in person by a clinical research coordinator prior to completing any study measures. Caregivers signed consent forms for their child’s participation, while all patients aged 7 years and older signed assent forms. Study recruitment was conducted by clinical research coordinators and principal investigator (JAH). Randomization and assessment were conducted and intervention fidelity was assessed by the clinical research coordinators.

### Statistical Methods

We performed a power analysis when designing the study. For simplicity, we used a 2-sided paired *t* test at a Bonferroni corrected 𝛼=.025. With 50 participants, we have at least 80% power to detect a difference of .45 standard deviations. Categorical data are summarized as frequency and percentage and continuous data as median and IQR. Study groups (young adults vs children, groups with different orders of interventions, and diagnosis groups) were compared using chi-square or Fisher exact tests for categorical variables and Mann-Whitney or Kruskal-Wallis tests for continuous variables. Paired data pre- versus postintervention and GI versus VR were analyzed using a Wilcoxon signed-rank test. Pearson correlations assessed relationships between continuous variables such as pain and anxiety. Statistical software used included SAS (version 9.4, SAS Institute Inc), SPSS (version 26, IBM Corp), and R (version 3.6.0, R Foundation for Statistical Computing). Unadjusted *P*<.05 was considered statistically significant.

## Results

### Demographics

Participant demographics are presented in Table 1. A total of 50 participants completed both interventions and were included in the analyses. The median time from diagnosis to study participation was 2.1 (IQR 0.2-8.1) years. There were no differences in randomization groups by participant age, gender, race, type of procedure, or household income. Participant age and gender did not differ across the 3 diagnostic groups (ie, cancer, sickle cell disease, other). Race (*P*<.001) and type of procedure (*P*<.001) were significantly different with a larger percentage of White individuals and port access procedures in participants with cancer and a larger percentage of African Americans and venipuncture procedures in participants with sickle cell disease. These results were expected and reflect racial differences in risk of disease and differences in standard treatment. Nearly all participants (47/50, 94%) engaged in active play during the VR intervention.

**Table 1:** Participant demographics (n=50).

		Value
Male, n (%)		26 (52)
Age (years), median (IQR)		13 (11-16)
**Race, n (%)**		
	White	26 (52)
	Black	18 (36)
	Other	6 (12)
**Procedure, n (%)**		
	Venipuncture	13 (26)
	Port access	26 (52)
	Dressing change	11 (22)
**Diagnosis, n (%)**		
	Cancer	31 (62)
	Sickle cell disease	12 (24)
	Other	7 (14)
**Parent education, n (%)**		
	High school	13 (26)
	Some college	16 (32)
	Bachelor degree	7 (14)
	Graduate degree	5 (10)
	Unknown	9 (18)
**Household income (US $), n (%)**		
	<25,000	8 (16)
	25,000-49,999	9 (18)
	50,000-74,999	6 (12)
	75,000-99,999	3 (6)
	>100,000	9 (18)
	Unknown	15 (30)

### Procedural Pain Outcomes

Self-reported pain scores on the VAS ranged from 0 to 100 across interventions. Scores for worst pain, average pain, nervousness about pain, and time spent thinking about pain did not differ between GI and VR. Similarly, there were no significant differences between interventions in CEMS score. There were no differences between the pain ratings of children and young adults in either intervention.

### State Anxiety Outcomes

The preprocedure state anxiety scores did not differ between GI and VR (median z-scores –0.38 vs –0.34, respectively, *P*=.24), nor did postprocedure state anxiety scores (median z-scores –0.53 vs –0.69, respectively, *P*=.44). When comparing the change from pre- to postprocedure, there was a significant decline in state anxiety reported for the VR intervention (median z-scores –0.34 vs –0.69, *P*<.001) and no significant change in the GI intervention (*P*=.07). There were no differences between children and young adults in state anxiety scores in either the GI or VR intervention.

### Relationship Between Procedural Pain and State Anxiety

In the GI intervention, there was a significant relationship between preprocedure state anxiety and nervousness about pain and time spent thinking about pain but not worst pain or average pain (Table 2). Postprocedure state anxiety following the GI intervention was significantly related to all areas of self-reported pain, including worst pain, average pain, nervousness about pain, and time spent thinking about pain. In the VR intervention, ratings of preprocedure state anxiety were significantly related to worst pain, average pain, nervousness about pain, and time spent thinking about pain (Table 2). Similarly, in the VR intervention, ratings of postprocedure state anxiety were significantly related to worst pain, nervousness about pain, and time spent thinking about pain. Following the VR intervention, state anxiety was no longer related to ratings of average pain.

**Table 2:** Relationship between pre- and postprocedural pain and state anxiety.

		State anxiety				
		GI^a^			VR^b^	
		Pre	Post	Pre		Post
**Worst pain**						
	*r^c^*	0.28	0.46	0.47		0.33
	*P* value	.05	<.001	<.001		.02
**Average pain**						
	*r*	0.18	0.31	0.49		0.26
	*P* value	.20	.03	<.001		.07
**Nervousness about pain**						
	*r*	0.45	0.38	0.48		0.40
	*P* value	.001	.01	<.001		.004
**Time spent thinking about pain**						
	*r*	0.45	0.38	0.56		0.51
	*P* value	.001	.01	<.001		<.001

^a^GI: guided imagery.

^b^VR: virtual reality.

^c^*r*: estimate of Pearson product-moment correlation coefficient.

### Impact of Pain Catastrophizing on Procedural Pain and Anxiety

Of the participants, 14% (7/50) had an elevated total pain catastrophizing score (>30). Greater levels of pain catastrophizing were associated with worst pain experienced during the procedure for both interventions (Table 3). Increased helplessness was associated with worst pain for participants in the GI intervention but not the VR intervention. Rumination and magnification were not related to worst pain.

Greater levels of pain catastrophizing were associated with higher average pain experienced during the procedure for both interventions (Table 3). Increased magnification and helplessness were associated with higher average pain for participants in both interventions, whereas rumination was not related to average pain in either intervention.

Greater levels of pain catastrophizing were associated with more nervousness about experiencing pain in the GI but not the VR intervention (Table 3). Increased rumination and helplessness were associated with more nervousness for participants in the GI but not the VR intervention. Magnification was not related to nervousness about pain in either intervention.

**Table 3:** Relationship between procedural pain and pain catastrophizing.

			Rumination				Magnification				Helplessness					PCS^a^ total		
			GI^b^		VR^c^		GI		VR		GI		VR		GI			VR
**Worst pain**																		
	*r^d^*	0.22		0.26		0.22		0.28		0.33		0.25		0.3			0.29	
	*P* value	.13		.07		.13		.06		.02		.09		.04			.04	
**Average pain**																		
	*r*	0.28		0.25		0.32		0.4		0.4		0.37		0.39			0.38	
	*P* value	.06		.09		.03		.005		.005		.01		.007			.009	
**Nervousness about pain**																		
	*r*	0.29		0.05		0.21		0.11		0.35		0.08		0.34			0.09	
	*P* value	.05		.72		.16		.46		.02		.60		.02			.56	
**Time spent thinking about pain**																		
	*r*	0.17		0.08		0.07		0.16		0.16		0.21		0.16			0.17	
	*P* value	.26		.60		.63		.29		.27		.15		.27			.24	
**CEMS^e^**																		
	*r*	0.16		0.25		0.18		0.21		0.22		0.17		0.22			0.23	
	*P* value	.28		.08		.21		.15		.14		.26		.14			.11	

^a^PCS: Pain Catastrophizing Scale.

^b^GI: guided imagery.

^c^VR: virtual reality.

^d^*r*: estimate of Pearson product-moment correlation coefficient.

^e^CEMS: Children’s Emotional Manifestation Scale.

There was no relationship between pain catastrophizing and time spent thinking about pain in either intervention (Table 3). There was no relationship between pain catastrophizing and CEMS ratings of pain in either intervention (Table 3). There was no relationship between pain catastrophizing and pre- or postprocedure state anxiety (Table 4). There was a significant relationship between pain catastrophizing and trait anxiety (*r*=0.44, *P*=.002).

**Table 4:** Relationship between anxiety and pain catastrophizing.

		Rumination			Magnification			Helplessness			PCS^a^ total	
		GI^b^	VR^c^	GI		VR	GI		VR	GI		VR
**Preprocedure state anxiety**												
	*r^d^*	0.16	0.18	0.20		0.10	0.21		0.14	0.22		0.16
	*P* value	.27	.24	.18		.51	.15		.36	.14		.28
**Postprocedure state anxiety**												
	*r*	0.11	0.09	0.14		0.05	0.27		0.23	0.21		0.16
	*P* value	.47	.56	.33		.73	.07		.12	.15		.28

^a^PCS: Pain Catastrophizing Scale.

^b^GI: guided imagery.

^c^VR: virtual reality.

^d^*r*: estimate of Pearson product-moment correlation coefficient.

### Impact of Trait Anxiety on Procedural Pain and Anxiety

There was a significant relationship between trait anxiety and all areas of self-reported procedural pain in the VR intervention (Table 5). In the GI intervention, trait anxiety was significantly related to worst pain and nervousness about pain but not average pain or time spent thinking about pain (Table 5). There was a significant relationship between trait anxiety and CEMS score during the GI but not the VR intervention (Table 5).

During both interventions, higher levels of trait anxiety were significantly correlated with higher pre- and postprocedure measures of state anxiety (GI preprocedure: *r*=0.58, *P*<.001; GI postprocedure: *r*=0.43, *P*=.002; VR preprocedure: *r*=0.42, *P*=.003; VR postprocedure: *r*=0.37, *P*=.01).

**Table 5:** Relationship between procedural pain and anxiety.

			Trait anxiety	
			GI^a^	VR^b^
**Worst pain**				
	*r^c^*	0.30		0.43
	*P* value	.03		.002
**Average pain**				
	*r*	0.24		0.48
	*P* value	.09		<.001
**Nervousness about pain**				
	*r*	0.30		0.51
	*P* value	.04		<.001
**Time spent thinking about pain**				
	*r*	0.10		0.51
	*P* value	.48		<.001
**CEMS^d^**				
	*r*	0.41		0.21
	*P* value	.003		.14

^a^GI: guided imagery.

^b^VR: virtual reality.

^c^*r*: estimate of Pearson product-moment correlation coefficient.

^d^CEMS: Children’s Emotional Manifestation Scale.

### Disease Group Differences

When participating in GI, there were no differences between diagnostic groups in procedure pain scores on the VAS. When participating in VR, there were no differences between diagnostic groups in worst pain (*P*=.61), average pain (*P*=.57), time spent thinking about pain (*P*=.27), or CEMS score (*P*=.70). There were, however, significant differences between diagnostic groups in the level of nervousness reported during the VR intervention (*P*=.04). Pairwise comparisons revealed that the significant differences were between cancer and other diagnoses (median scores 12 vs 0, *P*=.01), with more nervousness reported by those with cancer. There were no differences between cancer and sickle cell (*P*=.43) or sickle cell and other (*P*=.13). There were no significant differences between the diagnostic groups on the PCS total score or the PCS subscales.

Across interventions, there were no pre- or postprocedure anxiety score differences for patients in any disease group. The change of pre- to postanxiety scores differed significantly in the sickle cell disease group with greater declines in anxiety during VR compared to GI (median change in z-scores –0.14 vs 0.07, *P*=.03). Trait anxiety was comparable across diagnoses (*P*=.53).

## Discussion

### Principal Findings

Poorly managed procedure-related pain is acutely distressing and can lead to increased pain sensitivity throughout the lifespan, lowering the likelihood of seeking medical care as an adult [3]. This RCT was designed to identify low-risk, nonpharmacological options to manage pain and distress experienced during recurrent procedures where sedation is unwarranted. Specifically, we compared a highly effective distraction strategy, GI, with a less well known but more immersive strategy, VR. In general, we found the interventions performed similarly in their management of procedural pain. Across interventions, the majority of participants rated the procedures as causing a low level of pain; however, there was a subset of participants who self-reported high levels of pain, demonstrating the need to identify patients at higher risk of pain and distress.

Similar to previous literature [30-32], we found that the subgroup that held unhelpful beliefs about pain (ie, those with high pain catastrophizing scores) reported higher levels of worst pain and average pain across both interventions. Confirming our hypothesis, those with high pain catastrophizing reported experiencing less nervousness about pain during procedures that used VR than those using GI. Our findings suggest that the mechanism by which VR lessened the impact of pain catastrophizing on the experience of pain was twofold—by acting on feelings of helplessness and rumination about pain. We attribute these findings to the more immersive nature of VR, which increases the cognitive distraction to pain [35].

We had anticipated that those with more anxiety, whether state or trait, would have a more powerful response to VR. We did not expect there to be differences between how those with high state and trait anxiety responded to the interventions. State anxiety declined pre- to postprocedure during the VR intervention, specifically in participants who started with higher state anxiety. State anxiety is transient and situational. A good distractor consumes most of one’s cognitive energies, leaving little capacity to process pain and anxiety [42]. It makes sense, then, that those participants who were highly anxious about the procedure had a powerful response to the immersive nature of VR.

As a more global and stable construct, those with high trait anxiety responded differently. Contrary to our hypothesis, GI disrupted the relationships between trait anxiety and the variables of average pain and time spent thinking about pain but VR did not. Previous research has suggested that those with high trait anxiety experience greater increases in physiological arousal when presented with stress and more accurately perceive these changes compared to those with low trait anxiety [43,44]. The GI intervention offered 2 components that may have been a better fit for those with high trait anxiety: brief guidance in diaphragmatic breathing to reduce physiological arousal and the ability to watch the procedure. This finding suggests that VR may be more efficacious for children who are generally well adjusted but evidence a high degree of distress around a procedure, whereas GI may be more efficacious for those who have preexisting or chronic anxiety.

All study participants, regardless of their diagnosis, had a similar pain response during GI. Children and young adults with cancer, however, were more nervous about experiencing pain during the VR intervention than those in the other category. This may be a function of length of disease or procedure type (ie, port access vs dressing change). Participants with sickle cell disease responded with a more powerful reduction in anxiety when using VR than GI. While it is unclear why, we know that children and adolescents with sickle cell disease have a different pain trajectory than those with other diseases [45]. The transition from acute and intermittent vaso-occlusive pain crises in childhood to chronic pain in adolescence is well documented but remains poorly understood [46]. There were no differences in trait anxiety or beliefs about pain by diagnosis.

### Limitations and Future Research

This RCT crossover study compared 2 distraction interventions used during unsedated procedures in a real-world pediatric medical population. Although this study has several strengths, limitations must be noted. First, the selected procedures elicited low levels of pain and anxiety, which may have diluted differences between the interventions. Future research that includes more painful or frightening procedures, such as lumbar puncture or nasogastric tube placement, would advance the findings of this study. Had we known that the procedures in this study were so well tolerated for most participants, we would have screened for distress and excluded those who did not meet a minimum threshold.

A second limitation of this study is the lack of a control intervention. We determined that providing supportive distraction is the standard of care during procedures at our institution; therefore, we did not feel it was ethical to withhold. However, a control intervention with marginal distraction, such as engaging in conversation or encouraging the child to watch television, could have been used and would have served as a useful comparison to better elucidate the benefits of more distracting interventions such as GI and VR.

A third limitation of this study is the heterogeneity of the sample selected, including different diagnoses and procedures. While this yielded more efficient recruitment and realistic representation of children seen in a hematology, oncology, and blood and marrow transplant setting, it also introduced confounding variables that threatened the internal validity of the study. Future research should include either a more homogenous sample (eg, only children with sickle cell disease, only children with high pain catastrophizing) or a larger number of participants such that subgroups can be examined with greater confidence and a smaller margin of error.

### Clinical Implications and Conclusion

This study shows that, in general, VR works as well as GI to manage the pain and distress associated with common procedures experienced by children with an acute or chronic illness. We found that children who are primed for pain, based on beliefs about pain or because of their history of chronic pain, have a better response to VR. GI is a better intervention for those with high trait anxiety who may benefit from a greater sense of control when able to watch the procedure. As medical treatments are increasingly tailored at the individual level, mental health providers too need to give more thought to the power of individualized interventions. This new information advances our understanding of who may benefit more from GI and VR.

## Appendix

Multimedia Appendix 1 CONSORT-eHEALTH checklist (V 1.6.1).

## Abbreviations

CEMS: Children’s Emotional Manifestation Scale
CONSORT: Consolidated Standards on Reporting Trials
GI: guided imagery
PCS: Pain Catastrophizing Scale
RCT: randomized controlled trial
STAI: State-Trait Anxiety Inventory
VAS: visual analog scale
VR: virtual reality
